# Slow Transit Constipation: Pathophysiological Perspectives and Management Updates

**DOI:** 10.1111/1751-2980.70030

**Published:** 2026-02-06

**Authors:** Athanasios Syllaios, Stavros P. Papadakos, Alexandros Ioannou, Maximos Frountzas, Dimosthenis Michelakis, Dimitrios Patsouras, Spyridon Dritsas, Manousos‐Georgios Pramateftakis, Dimitrios Schizas

**Affiliations:** ^1^ First Department of Surgery National and Kapodistrian University of Athens, Laiko General Hospital Athens Greece; ^2^ Colorectal Department, Addenbrooke's Hospital Cambridge University Hospitals NHS Trust Cambridge United Kingdom; ^3^ First Department of Gastroenterology National and Kapodistrian University of Athens, Laiko General Hospital Athens Greece; ^4^ Department of Gastroenterology Alexandra General Hospital Athens Greece; ^5^ Department of Colorectal Surgery Royal Marsden NHS Foundation Trust London United Kingdom; ^6^ Department of Surgical Oncology, University General Hospital of Heraklion University of Crete Heraklion Greece; ^7^ Second Department of Propaedeutic Surgery National and Kapodistrian University of Athens, Laiko General Hospital Athens Greece; ^8^ First Department of Surgery Evangelismos General Hospital Athens Greece; ^9^ Fourth Department of Surgery Aristotle University of Thessaloniki, “George Papanikolaou” General Hospital Thessaloniki Greece

**Keywords:** antegrade continence enema, colonic motility, refractory constipation, slow transit constipation, subtotal colectomy

## Abstract

Slow transit constipation (STC) is a chronic colonic motility disorder characterized by markedly delayed transit, leading to reduced bowel movements, abdominal discomfort, and significant quality‐of‐life impairment. It predominantly affects women and is associated with abnormalities in enteric neuronal signaling, smooth muscle contractility, interstitial cells of Cajal, gut peptides, bile acid homeostasis, and autonomic regulation. Secondary causes of constipation and structural lesions must be excluded before the diagnosis of STC, with colonic transit studies serving as the gold standard. Complementary investigations such as anorectal manometry and defecography help detect coexisting outlet obstruction, which can alter management. The treatment of STC should follow a stepwise approach, beginning with dietary and lifestyle modification, osmotic and stimulant laxatives, and prokinetics such as prucalopride. Secretagogues and bile acid modulators may offer additional benefit. Biofeedback therapy is primarily indicated for overlapping dyssynergic defecation. For refractory STC, interventional therapies, such as fecal microbiota transplantation, acupuncture, sacral nerve stimulation, and transanal irrigation, are found to have equivocal outcomes. Antegrade continence enema procedures can be an alternative for patients unsuitable for colectomy. Surgical options, including subtotal colectomy with ileosigmoid or cecorectal anastomosis, and total colectomy with ileorectal anastomosis, are reserved for carefully selected patients with medically intractable symptoms, following thorough physiological evaluation. Although advances in understanding STC pathophysiology are guiding novel therapeutic development, robust randomized controlled trials remain scarce. Optimal care requires multidisciplinary collaboration between gastroenterologists, colorectal surgeons, and pelvic floor specialists to ensure accurate diagnosis, tailored treatment, and improved long‐term outcomes.

## Graphical abstract

Slow transit constipation (STC) is a complex neuromuscular disorder driven by interstitial cells of Cajal (ICCs) loss and enteric neuropathy. Diagnosis relies on objective transit testing while excluding pelvic floor dysfunction. Management follows a stepwise, phenotype‐driven approach, progressing from conventional laxatives to emerging targeted therapies and selective surgery for refractory cases. 3D‐HDAM, three‐dimensional high‐definition anorectal manometry; FMT, fecal microbiota transplanation; HR‐ARM, high‐resolution anorectal manometry; RCTs, randomized controlled trials; SNS, sacral nerve stimulation; TAI, transanal irrigation.

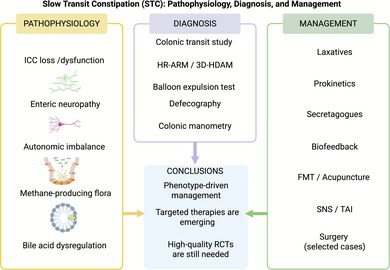

## 1 Introduction

Chronic constipation is one of the most prevalent gastrointestinal (GI) complaints worldwide, with population‐based studies estimating rates between 10% and 20% in Western countries and even higher—up to 30%—among the elderly [1, 2]. Chronic constipation exhibits a significant female predominance, with women being affected up to twofold over men. In addition, its prevalence also increases with advancing age, institutionalization, and polypharmacy [3]. Beyond its frequency, constipation carries a substantial psychological and economic burden, as it accounts for millions of outpatient visits annually [4] and drives significant over‐the‐counter laxative use [1, 5]. Its etiologic spectrum spans secondary causes, including anatomical (anorectal or colonic diseases), metabolic (diabetes mellitus, hypercalcemia, etc.), endocrine (hypothyroidism), neurological (Parkinson's disease, spinal cord lesions, etc.), and iatrogenic factors such as opioids, calcium‐channel blockers, and anticholinergics, and primary functional disorders [6, 7]. Even medications such as glucagon‐like peptide receptor agonists (GLP‐1RAs), despite their widespread utility in diabetes mellitus and obesity management, affect GI motility and are frequently associated with adverse events including constipation, diarrhea, and nausea [8]. Functional constipation and related constipation disorders are categorized according to the Rome IV criteria into functional constipation, irritable bowel syndrome with constipation, opioid‐induced constipation, and functional defecation disorders [9, 10]. From a pathophysiological perspective, constipation is commonly further subclassified into normal‐transit constipation (NTC), slow transit constipation (STC), and defecatory disorders, a framework widely used to guide diagnostic evaluation and management.

Among these entities, STC represents a particularly challenging subtype for diagnosis in clinical practice [11]. First proposed by Proton and Lennard‐Joners in 1986, STC is now recognized as a form of chronic constipation [12] characterized by a significant delay in the passage of stool through the colon once over 3 days, or even once every 6 to 10 days, a blunted or absent urge to defecate, and resistance to dietary fiber and conventional laxatives [13]. Physiological studies have demonstrated that patients with STC often have a reduction in the frequency of high‐amplitude propagated contractions (HAPCs), an attenuated gastrocolonic response, and evidence of colonic neuromuscular dysfunction ranging from enteric neuropathy and myopathy to decreased numbers of interstitial cells of Cajal (ICCs), which act as the colonic pacemaker cells [14]. Epidemiologically, STC is less common than NTC but disproportionately presents in tertiary motility clinics, comprising 15%–30% of all chronic constipation cases referred for specialized evaluation [15]. Notably, up to 60% of women with refractory constipation show delayed colonic transit on objective testing [16]. Pediatric cases with STC are rare; while in adult cohorts, it predominantly affects young and middle‐aged women, suggesting a potential hormonal or connective tissue component [17].

Diagnosis of STC requires objective demonstration of delayed transit, most often through radiopaque marker studies (Sitzmarks test), scintigraphy, or the increasingly adopted wireless motility capsule (WMC) test. However, interpretation is nuanced—as many as 60% of patients with dyssynergic defecation can also show delayed marker retention [18], underscoring the need to exclude pelvic floor dysfunction with anorectal manometry and balloon expulsion testing before labeling a patient with STC. Management of STC remains complex and is often frustrating for patients and clinicians alike. Novel pharmacological therapies, such as secretagogues (lubiprostone, linaclotide, plecanatide) and serotonergic prokinetics like prucalopride, can enhance colonic motility and improve stool frequency; however, many patients only achieve partial relief [6]. In carefully selected, truly refractory cases—particularly those with manometric or histologic evidence of colonic neuropathy**—**total colectomy with ileorectal anastomosis may offer symptomatic benefit, though at the cost of significant morbidity and variable long‐term outcomes [19].

This narrative review emphasizes emerging mechanistic insights involving the gut microbiome–bile acid–motility axis, advances in high‐resolution physiological testing, and the expanding but heterogeneous evidence base for non‐pharmacological interventions of STC. By integrating recent translational and clinical data, we aim to refine current therapeutic algorithms and highlight priorities for future research in this challenging disorder.

## 2 Pathogenesis of STC

GI peristalsis is regulated by a highly integrated and evolutionarily conserved network of motor control systems that collectively ensure the propulsion and mixing of luminal contents [20]. This complex process is not attributable to a single dominant mechanism but rather reflects the coordinated interplay between intrinsic and extrinsic neural pathways, smooth muscle contractile properties, specialized pacemaker ICCs, and modulatory endocrine and paracrine signals [11].

### 2.1 Colonic Dysmotility

Colonic motor activity is a highly coordinated process that varies temporally and spatially, influenced by factors such as diet habits, sleep, physical and emotional stress, gender, and aging [21]. In STC cases, there is a significant reduction in overall colonic motor activity, particularly in the frequency of HAPCs, which are essential for propelling stool toward the rectum [22]. These contractions occur less frequently, exhibit reduced amplitude, and often terminate prematurely in STC patients [22]. The gastrocolonic response, a surge in motility triggered by food intake, is significantly diminished or absent, as is the increased motor activity typically observed upon waking [23]. Despite these impairments, the diurnal rhythm of colonic activity remains preserved [24]. In addition, periodic rectal motor activity, characterized by rhythmic contractions in the rectosigmoid region at approximately 3 cycles per min, is significantly increased in STC patients, particularly at night, potentially acting as a “nocturnal brake” that further impedes stool transit [25].

The complexity of gut peristalsis arises from multiple overlapping mechanisms [20]. ICCs serve as the primary pacemaker cells, generating slow waves that rhythmically depolarize smooth muscle cells, bringing them close to the threshold for calcium influx and subsequent contraction [26]. In STC cases, propagation of these slow waves is impaired, with reduced velocity and disrupted coordination, which subsequently contributes to diminished HAPCs [27]. Slow waves typically propagate as broad wavefronts along the colon, with near‐simultaneous circumferential activation and slower longitudinal progression [28]. Such propagation is often disorganized, with ectopic pacemaker activity or collisions between slow waves further disrupting coordinated motility [27]. Smooth muscle stretch, mediated by mechanosensitive ion channels, can also trigger depolarization and rhythmic contractions; however, it is attenuated in STC. Moreover, the migrating motor complex (MMC), a cyclical pattern of neural‐driven contractions during fasting [29], is disrupted, with reduced propagation of the excitatory band that drives slow wave‐associated peristalsis [30, 31]. These combined deficits in pacemaker activity, neural coordination, and muscle responsiveness result in the sluggish and ineffective colonic propulsion characteristic of STC.

### 2.2 Neural and Structural Abnormalities

Neurological and structural alterations are central to the pathophysiology of STC. A hallmark finding in STC is a significant dysfunction or loss of ICCs in both the submucosal and muscular layers of the colon [26, 32]. ICCs form an intricate syncytial network with smooth muscle cells and platelet‐derived growth factor receptor α‐positive (PDGFRα^+^) cells, enabling finely tuned communication between the enteric nervous system, muscularis propria, and local paracrine signaling pathways [33]. Loss of ICCs disrupts this electro‐mechanical integration, leading to impaired generation and propagation of slow waves, which in turn compromises coordinated motility [33]. Emerging insights also highlight that ICCs are not homogeneous subtypes, such as myenteric ICC, which drive pacemaker activity, and intramuscular ICC, which mediate neurotransmission, may be differentially affected in STC [34]. Structural degeneration of ICC networks, coupled with mitochondrial abnormalities within ICCs, likely further compromises their pacemaker capacity by impairing energy supply for slow wave generation. Collectively, these overlapping neural and structural defects establish a disturbance of the neuromuscular system of the colon, which might explain the reason why motility in STC remains refractory to conventional laxatives, suggesting that therapies aimed at restoring ICC networks or enteric neuronal integrity could hold future promise.

Further structural abnormalities include a reduction in myenteric ganglion cells—the intrinsic neuronal hubs of the enteric nervous system—which has been consistently observed in colectomy‐obtained specimens from STC patients [35]. This neuronal loss diminishes excitatory and inhibitory signaling balance, contributing to ineffective colonic propulsion. Notably, it remains unclear whether these cellular alterations are a primary features of STC pathogenesis or if they develop secondarily to chronic exposure to factors such as stimulant laxatives, medications, or long‐standing bowel stasis [20]. As mentioned above, STC patients often exhibit blunted colonic reflexes and an increase in retrograde propulsion, both of which aggravate stasis of fecal matter [20]. Moreover, autonomic dysregulation has been increasingly recognized as a contributor, with increased sympathetic activation and disturbed parasympathetic function documented in irrtable bowel syndrome with predominant constipation (IBS‐C), leading to reduced colonic contractions in response to stimuli such as balloon distension or prokinetic agents like bisacodyl [36].

### 2.3 Neurotransmitter and Hormonal Dysregulation

Neurochemical and endocrine disturbances represent a significant dimension of STC pathophysiology [37]. While stool hardness in chronic constipation has been attributed to excessive water absorption, colonic absorptive function appears to be largely preserved in most patients. This suggests that factors beyond epithelial transport, particularly neurohormonal modulation of motility, may be the key drivers of delayed transit [38].

Epidemiological observation shows a higher prevalence of STC in women, particularly in young and middle‐aged adults, raising the possibility of sex hormone‐related influences on colonic neuromuscular activity [39]. Though reductions in ovarian or adrenal hormones have been proposed, routine estrogen and progesterone levels in affected individuals are typically within normal limits. The relationship between menstrual cycle phases and colonic transit remains inconsistent, with studies reporting both slowing and unaltered transit during the luteal phase [40]. Mechanistic evidence from colectomy specimens has demonstrated a downregulation of progesterone‐dependent contractile G proteins alongside upregulation of inhibitory G proteins, possibly due to overexpression of progesterone receptors in colonic smooth muscle [41, 42]. These findings provide a molecular rationale for the female predominance observed in STC.

Alterations in neurotransmitters in STC have been variably reported. Reductions in vasoactive intestinal peptide (VIP) have been documented, but their causal role remains uncertain [43]. Of particular interest is serotonin (5‐hydroxytryptamine [5‐HT]), a key modulator of GI motility synthesized predominantly in mucosal enterochromaffin cells [44]. Mechanical or chemical stimulation of the colonic mucosa triggers the release of 5‐HT and other mediators such as calcitonin gene‐related peptides, which activate intrinsic primary afferent neurons in the myenteric plexus. This initiates the peristaltic reflex via ascending excitatory pathways—mediated by acetylcholine and substance P, inducing smooth muscle contraction—and descending inhibitory pathways, which involve nitric oxide, VIP, and adenosine triphosphate (ATP), resulting in segmental relaxation [44].

In patients with STC, both the gastrocolonic response and the ascending contraction component of the peristaltic reflex are blunted, implicating serotonergic signaling defects [44]. This is supported by pharmacological evidence, showing that 5‐HT_4_ receptor agonists (e.g., tegaserod, prucalopride) enhance both ascending contraction and descending relaxation and accelerate intestinal transit, whereas 5‐HT_3_ antagonists (e.g., granisetron) attenuate these responses [45]. Whether these serotonergic abnormalities stem from reduced serotonin availability, altered receptor density, or dysfunctional serotonin reuptake remains unresolved [44].

### 2.4 Colonic Flora and Methane Production

Gut microbiota, particularly methanogenic flora like *Methanobrevibacter smithii* , are increasingly recognized in the pathogenesis of STC [46, 47]. Methane, an inert gas produced by certain microbes, is more abundant in constipated individuals and may directly impair colonic muscle contractions. Patients with methanogenic flora exhibit impaired colonic motility and increased rectal hypersensitivity, though small‐bowel motility remains unaffected [47]. The lower colonic pH value in these individuals may further influence motility. Whether methanogenic flora is a cause or consequence of STC requires further investigation; however, its presence is associated with more severe symptoms, particularly in women.

### 2.5 Fluid and Electrolyte Transport

Fluid and electrolyte handling by the colon plays a pivotal role in determining stool consistency and transit, although disturbances in these processes are not considered the primary driver of STC [48]. On average, approximately 9 L of fluid enter the GI tract daily, with about 1 L of the residual fluid reaching the colon, resulting in a stool water content of roughly 200 mL [49]. Under normal circumstances, the colon has substantial absorptive reserve, capable of reabsorbing up to 4 times its usual daily volume if required, provided that intraluminal flow is not excessively rapid [49]. Colonic water and ion transport is regulated by the following mechanisms:

- Coupled sodium chloride absorption occurs throughout the small and large intestine via coordinated activity of apical Na^+^/H^+^ exchangers (NHE2, NHE3) and Cl^−^/HCO_3_^−^ exchangers (SLC26A3, SLC26A6) [50]. Once inside the epithelial cells, sodium is pumped into the bloodstream through the basolateral Na^+^/K^+^ ATPase, while chloride is transported out through potassium–chloride cotransport systems. This process is modulated by hormonal and neurohumoral signals. For example, absorption is generally inhibited by cyclic adenosine monophosphate (cAMP)‐ or Ca^2+^‐elevating mediators, while it is enhanced epidermal growth factor and tyrosine kinase activation [50].
- Electrogenic Na^+^ absorption is mediated mainly by epithelial sodium channels (ENaC) in the distal colon, allowing sodium entry without simultaneous chloride uptake [51]. ENaC activity is stimulated by aldosterone and glucocorticoids and influenced by intracellular signaling cascades.
- Chloride secretion is driven largely by crypt epithelial cells via cystic fibrosis transmembrane conductance regulator (CFTR) and Ca^2+^‐activated chloride channels [49]. Chloride is brought into the cells from the basolateral side through the Na^+^/K^+^/Cl^−^ cotransporter (NKCC1). The energy for this process is maintained by extrusion of Na^+^ through the Na^+^/K^+^‐ATPase and potassium recycling through basolateral K^+^ channels [49]. Guanylate cyclase‐C (GCC) receptor agonists, such as linaclotide and plecanatide, increase intracellular cyclic guanosine monophosphate (cGMP), which stimulates CFTR activity and promotes secretion.

**FIGURE 1:**
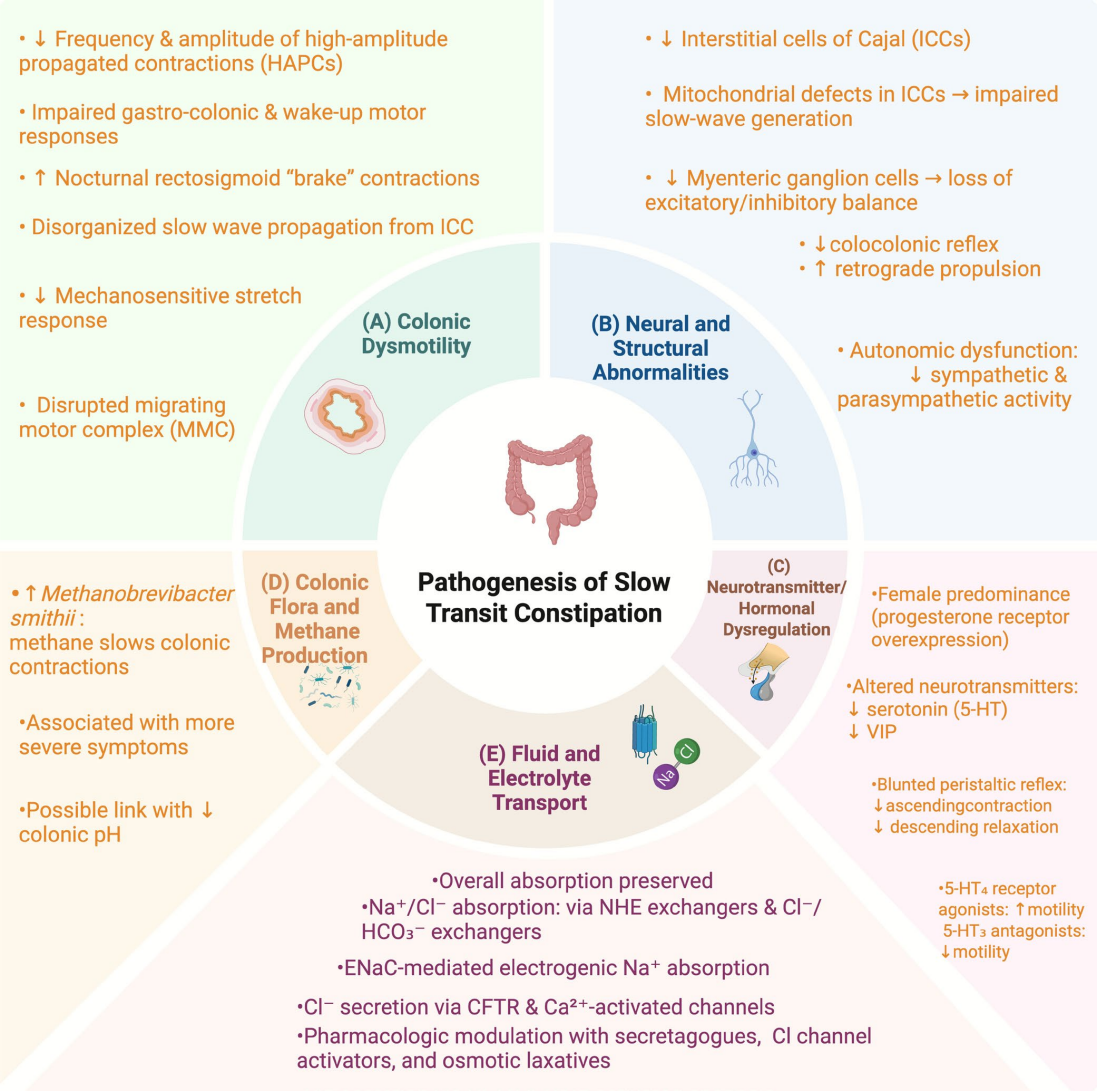
Multifactorial pathophysiology of slow transit constipation (STC). (A) Colonic dysmotility: reduced/amplitude‐impaired high‐amplitude propagated contractions (HAPCs), blunted gastrocolonic and wake‐up responses, disorganized interstitial cells of Cajal (ICCs)‐driven slow wave propagation, and disrupted migrating motor complex. (B) Neural and structural defects: loss/degeneration of ICCs, mitochondrial abnormalities, reduced myenteric neurons, and autonomic imbalance. (C) Neurotransmitter/hormonal dysregulation: altered progesterone and serotonergic signaling, reduced vasoactive intestinal peptide (VIP). (D) Microbiome: methanogenic overgrowth (e.g., *Methanobrevibacter smithii* ) producing transit‐slowing methane. (E) Fluid/electrolyte handling: subtle dysregulation of absorption/secretion influencing stool hydration. These mechanisms interact to produce refractory delayed transit. CFTR, cystic fibrosis transmembrane conductance regulator. Created with BioRender.com.

Pharmacological strategies have been designed to influence these transport processes to adjust stool water content and transit characteristics [48]. Agents such as lubiprostone (a chloride channel 2 [ClC‐2] activator) promote intestinal secretion and facilitate bowel movements. Secretagogues that increase chloride and bicarbonate release (e.g., GCC agonists) and osmotic laxatives that create osmotic gradients within the intestinal lumen can also enhance stool hydration and motility [48]. The equilibrium between intestinal absorption and secretion is evident in the actions of bile acids. When present in excessive amounts within the colon, they stimulate chloride and water release, resulting in diarrhea, whereas diminished bile acid entry tends to slow secretion and promote harder stool [52]. Dysregulation of NHE or other transport proteins may subtly shift this balance in STC, influencing stool consistency and transit dynamics. Pathogenesis of STC is briefly summarized in Figure 1.

Collectively, these findings support the concept that STC represents a multifactorial neuromuscular disorder rather than a single‐pathway motility defect. Recent advances have shifted the focus from isolated abnormalities in colonic contractility toward integrated dysfunction involving pacemaker cell networks, enteric neuronal signaling, bile acid‐dependent epithelial transport, and microbiota‐derived metabolites such as methane. This evolving mechanistic framework not only refines disease phenotyping but also provides a rationale for emerging targeted interventions, including microbiome modulation, bile acid‐based therapies, and strategies aimed at restoring neuromuscular integrity.

## 3 Diagnosis of STC

### 3.1 Initial Clinical Evaluation

Diagnostic assessment of STC should begin with a systematic approach to rule out secondary causes of constipation, characterize the symptom patterns, and identify potential coexisting disorders [48]. A comprehensive evaluation of patient's medical, surgical, and pharmacological history is essential, which should focus on systemic illnesses such as hypothyroidism, diabetes mellitus, Parkinson's disease, connective tissue disorders, and multiple sclerosis, as well as medications known to impair motility (e.g., opioids, anticholinergics, calcium channel blockers [6]).

Dietary and lifestyle evaluation should include daily fiber and fluid intake, physical activity, dietary patterns, and any habitual suppression of the urge to defecate [48]. Symptom characterization must address the onset, duration, severity, and the presence of alarming features such as hematochezia, unexplained anemia, involuntary weight loss, or a family history of colorectal cancer. Bowel habits—including stool frequency, consistency (according to Bristol Stool Form Scale), straining effort, and sense of incomplete evacuation—help establish a clinical profile [6].

Physical examination should include an abdominal evaluation for palpable stool masses, particularly in the left lower quadrant, and a careful digital rectal examination (DRE) [53]. DRE remains one of the most valuable tools of the initial evaluation, providing insight into anal sphincter tone, rectal compliance, and pelvic floor coordination during simulated evacuation. The findings such as paradoxical anal contraction, absence of perineal descent, or inadequate abdominal push effort should raise suspicion for dyssynergic defecation, which frequently coexists with STC and requires different management strategies [53]. Psychosocial factors should also be explored, as anxiety, depression, and emotional stress can influence the functions of the gut–brain axis, potentially aggravating symptoms [13]. Symptom questionnaires, stool diaries, and validated constipation scoring systems can standardize symptom assessment and facilitate longitudinal monitoring.

### 3.2 Specialized Diagnostic Testing

If symptoms of constipation persist despite initial lifestyle and dietary interventions, and secondary causes have been excluded, targeted physiological testing is warranted to confirm STC diagnosis and distinguish it from other constipation subtypes [49].

Studies on colonic transit are the cornerstone of constipation diagnosis [54]. The most widely used colonic transit testing is the radiopaque marker test, in which retained markers are quantified after a defined interval, typically 72 h [6]. In STC, retention is diffuse throughout the colon, in contrast to the rectosigmoid accumulation in outlet obstruction. Alternative methods include scintigraphic transit assessment and WMCs, which provide segmental transit time and additional small bowel motility information [49].

Anorectal manometry evaluates resting and squeeze pressures, rectoanal inhibitory reflex (RAIR) integrity, and coordination of rectoanal muscles during simulated defecation [55]. Conventional, non‐high‐resolution anorectal manometry (non‐HR‐ARM) is performed with catheters having 3 or 6 sensors positioned along or around the anal canal [56]. Because these sensors simultaneously cover the entire length of the high‐pressure zone, the catheter is inserted once and then remains stationary throughout the examination. Routine clinical testing therefore no longer requires a stepwise pull‐through maneuver, unlike older descriptions from decades ago or traditional esophageal manometry [56]. HR‐ARM, which was introduced in 2007, improved upon this by using catheters with multiple circumferential sensors distributed along the probe, allowing simultaneous pressure measurements across the rectum and anal canal without catheter movement. This offers greater spatial resolution, shorter testing time, and clearer assessment of coordination during defecation. More recently, 3‐dimensional high‐definition anorectal manometry (3D‐HD‐ARM) has enabled 3D spatiotemporal mapping, providing even more detailed insights into anorectal physiology [57]. HR‐ARM and 3D‐HDAM are now widely adopted in clinical practice, which progressively supersede traditional methods. Rectal balloon expulsion test is a simple, low‐cost outpatient tool that evaluates rectal evacuation by assessing the time required to expel a rectally inflated balloon, usually with 50 mL of warm water, in the seated position. A prolonged expulsion time, conventionally defined as over 1 min, suggests impaired evacuation and may indicate pelvic floor dysfunction [58]. Variability exists depending on the type of balloon used and the methodology of measurement, with some studies proposing shorter cut‐off values [59, 60], while others inflate the balloon to the point of perceived desire to defecate to account for altered rectal sensation [61]. Although the test cannot identify structural abnormalities, it has good reproducibility, correlates with findings from defecography and manometry, and has predictive value for response to biofeedback therapy [62].

Defecography, also known as evacuation proctography, is a fluoroscopic technique that evaluates anorectal anatomy and pelvic floor function during simulated defecation [63]. After rectal instillation of a thick barium paste, the patient evacuates while real‐time imaging captures changes in the anorectal angle, pelvic floor descent, and rectal morphology. It is mainly indicated in patients with refractory constipation, incomplete evacuation, suspected prolapse, or unexplained anorectal symptoms. Defecography can identify functional abnormalities, such as paradoxical contraction of the puborectalis muscle (dyssynergic defecation) or excessive pelvic floor descent, as well as structural disorders including rectocele, enterocele, sigmoidocele, intussusception, and rectal prolapse. While its main limitation is radiation exposure, defecography remains widely used due to its accessibility and ability to assess both the structure and function in a physiological setting. Magnetic resonance (MR) defecography avoids radiation but is less available and more costly [64]. Colonic manometry may be reserved for complex or refractory cases by assessing neuromuscular integrity and propagatig motor patterns [49]. In combination with barostat study in specialized motility centers, colonic manometry provides detailed assessment of colonic motor activity, tone, and sensory function in individuals with chronic constipation [65]. It involves the placement of a multisensor probe—traditionally solid‐state or water‐perfused—with newer high‐resolution fiber‐optic catheters offering improved spatial detail. Extended 24‐h ambulatory recording enables evaluation of natural colonic motility, postprandial responses, and reactions to pharmacological agents [65]. Key parameters include HAPCs, gastrocolonic and waking responses, and colonic tone/compliance. Distinct abnormal patterns can help differentiate myopathic from neuropathic STC and guide treatment decision‐making. Although technically demanding and available only in specialized centers, colonic manometry is considered safe and clinically valuable for tailoring management in severe constipation.

In select cases, biochemical testing for bile acid malabsorption or evaluation of gut microbiota composition may offer additional insights, as disturbances in bile acid signaling and microbial metabolites have been implicated in colonic motility regulation [49]. Histopathological examination of full‐thickness biopsy samples, though rarely required, can be used to confirm rare neuropathic or myopathic causes of severe dysmotility [66]. Collectively, the diagnosis of STC ultimately relies on objective demonstration of delayed colonic transit in the absence of outlet obstruction, interpreted within the context of a meticulous clinical evaluation. The abovementioned diagnostic tools are briefly summarized in Table 1.

**TABLE 1:** Diagnostic tools for slow‐transit constipation (STC).

Diagnostic tools	Objectives	Key findings in STC
Detailed medical history and physical examination	Identify potential secondary causes of constipation, assess symptom onset, dietary habits, and bowel patterns	Long‐standing symptoms, refractory to standard measures, absence of alarming features
Digital rectal examination	Evaluate sphincter tone, pelvic floor function, and detect structural abnormalities	Normal or reduced anal tone, absence of dyssynergia in isolated STC
Symptom questionnaires (e.g., constipation assessment scale, Bristol Stool Form Scale)	Quantify symptom severity, stool form, and defecation frequency	Hard stools (types 1–2), infrequent bowel movements
Colonoscopy	Rule out structural abnormalities in patients with alarming symptoms	Normal mucosa and anatomy in functional STC
Colonic transit studies (radiopaque marker test, scintigraphy, wireless motility capsule)	Measure colonic transit time and differentiate slow transit from normal or rapid transit	Prolonged colonic transit time > 72 h, diffuse marker retention
Balloon expulsion test	Screening for defecatory disorders	Normal expulsion time in isolated STC
Anorectal manometry	Assess rectoanal inhibitory reflex (RAIR), sphincter pressures, and coordination during defecation	Preserved RAIR, normal coordination in isolated STC
Defecography (conventional or magnetic resonance imaging)	Visualize anorectal anatomy and function during defecation	Normal structural findings in isolated STC
Colonic manometry	Evaluate colonic motor patterns, tone, and responses to stimuli	Reduced or absent high‐amplitude propagating contractions, blunted gastrocolonic response

## 4 Conventional Treatments for STC

Management of STC is guided by the chronicity of symptoms, underlying pathophysiology, and patient's response to initial measures [49], spanning from lifestyle and dietary optimization to targeted pharmacological and, in selected cases, procedural interventions. The American Neurogastroenterology and Motility Society and the European Society of Neurogastroenterology & Motility (ANMS‐ESNM) guidelines emphasize a stepwise strategy, starting with noninvasive measures and progressing to advanced therapies when conservative approaches fail [67].

### 4.1 Lifestyle and Dietary Modifications

#### 4.1.1 Fluid Intake

Although adequate hydration is generally encouraged, increasing fluid consumption alone rarely alleviates constipation in the absence of dehydration [68]. Evidence from controlled studies does not support the routine recommendation of high water intake as an isolated intervention [68]. However, adequate hydration remains important when increasing dietary fiber or using osmotic laxatives to avoid worsening stool hardness. Unfortunately, randomized controlled trials (RCTs) specifically addressing fluid intake in patients with STC are lacking, and current practice is mainly extrapolated from studies on patients with mixed or unclassified constipation.

#### 4.1.2 Dietary Fiber

Most guidelines recommend a daily fiber intake of 25–30 g for patients with constipation, particularly from soluble sources such as psyllium (ispaghula), oat bran, and pectin‐rich fruits [5]. Insoluble coarse fibers can act through mechanical stimulation of mucosal secretion, whereas soluble fibers primarily function via water‐holding capacity and fermentation to short‐chain fatty acids, which can promote motility [69, 70]. Therefore, in some patients—especially those with IBS‐C—symptoms may worsen [49]. In cases that cannot achieve symptom improvement with adequate fiber intake, other mechanisms should be considered, including dyssynergic defecation or markedly delayed transit [49].

#### 4.1.3 Physical Activity

Increased physical activity is not uniformly effective in STC, particularly in younger individuals with severe disease [48]. However, it may improve general GI function and well‐being. In elderly or physically inactive patients, incorporating exercise into rehabilitation programs may confer a modest benefit.

#### 4.1.4 Dietary Restrictions and Special Diets

Low‐fermentable, oligosaccharides, disaccharides, monosaccharides, and polyols (FODMAP) dietary patterns may alleviate symptoms in some patients with IBS‐C; however, evidence specific for isolated STC is insufficient [70]. These regimens should be individualized as excessive restriction may reduce dietary fiber and worsen constipation.

### 4.2 Pharmacological Therapy

Pharmacological treatments should be considered when lifestyle and dietary interventions do not provide sufficient relief [48]. Medications are typically selected based on their mechanisms of action and patient‐specific factors, with therapy often requiring dose titration.

#### 4.2.1 Osmotic Laxatives

Osmotic agents draw water into the intestinal lumen, increasing water content and fecal volume [49]. The efficacy and safety of polyethylene glycol (PEG) for constipation are supported by RCTs, showing superiority to lactulose in head‐to‐head comparisons [71, 72]. Lactulose, while safe, may cause bloating due to bacterial fermentation [5]. Magnesium oxide is also used in clinical practice but lacks robust evidence, which should be used in caution in elderly patients or those with renal impairment due to the risk of electrolyte disturbances [5, 73]. Similarly, long‐term use of phosphate salts lack strong evidence and is associated with significant risks of hyperphosphatemia and acute kidney injury (nephrocalcinosis), especially in elderly patients or those with impaired renal function [74].

#### 4.2.2 Stimulant Laxatives

Stimulant laxatives, such as bisacodyl, sodium picosulfate, and senna, stimulate intestinal secretion and motility via activation of the enteric nervous system and induction of prostaglandin release [5]. Clinical trials have demonstrated their efficacies in improving bowel frequency and associated symptoms without evidence of long‐term tolerance or colonic damage. Adverse events associated with the use of stimulant laxatives are typically limited to abdominal cramping and diarrhea [75].

#### 4.2.3 Prosecretory Agents

Lubiprostone is a highly selective ClC‐2 activator, enhancing chloride and fluid secretion in the small intestine and colon, which in turn facilitates stool passage [76]. The utility of lubiprostone leads to improvements in bowel movements, stool frequency, consistency, and associated symptoms, with nausea being the most common adverse event. While GCC receptor agonists such as linaclotide and plecanatide increase intracellular cyclic guanosinc monophosphate (cGMP) in intestinal epithelial cells, opening CFTR channels and promoting chloride and bicarbonate secretion [77]. Both agents demonstrate efficacy in improving stool frequency, consistency, and reducing straining, with diarrhea being the primary side effect.

#### 4.2.4 Serotonergic Agents

Prucalopride, a selective 5‐HT_4_ receptor agonist, enhances colonic motility through stimulation of enteric neurons [5]. A multicenter RCT has shown significant improvements in bowel movement frequency and symptom relief [78]. It is generally well tolerated, with headache, abdominal discomfort, and diarrhea being the most frequent adverse events.

### 4.3 Anorectal Biofeedback Therapy

For patients with coexistent dyssynergic defecation, biofeedback therapy is the treatment of choice [79]. It uses real‐time visual, auditory, or verbal feedback to retrain coordinated abdominal straining and pelvic floor relaxation during defecation. Standard protocols of the biofeedback therapy incorporate patient education, simulated evacuation training, and—in some cases—sensory retraining. Long‐term response rates to the biofeedback therapy of approximately 70% have been reported, and its utility is supported by the ANMS‐ESNM guideline recommendations [67].

In summary, initial management of STC should focus on lifestyle and dietary modification to ensure adequate fiber and fluid intake (tailored to individual tolerance) and maintain regular physical activity. When these modalities are insufficient, pharmacologic agents are recommended, beginning with osmotic and stimulant laxatives. Prosecretory and serotonergic agents offer effective options for refractory cases. And biofeedback therapy is the treatment of choice for coexisting dyssynergic defecation. Selection and sequencing of therapies should be individualized, aiming at symptom control, minimal adverse events, and preservation of quality of life (QoL).

## 5 Innovative Non‐Pharmacological Therapies

### 5.1 Fecal Microbiota Transplantation (FMT)

Alterations in gut microbiota composition and metabolic activity are increasingly recognized as contributors to colonic dysmotility in STC [80]. Ge et al. transplanted fecal microbiota collected from STC patients who had received effective FMT into pseudo‐germ‐free mice, which induced constipation‐like features such as reduced pellet frequency, lower fecal water content, delayed GI transit, and weaker colonic smooth muscle contractions [81]. In addition, levels of butyrate and secondary bile acids in the cecum of these mice, including deoxycholic acid (DCA) and lithocholic acid (LCA), were significantly reduced compared to those in mice transplanted from healthy donors. Supplementation with butyrate or DCA partially reversed these defects by enhancing fecal water content and restoring contractile amplitude/frequency. These findings demonstrate that STC‐associated dysbiosis can causally impair gut motility [81]. FMT seeks to restore a more physiologic microbial ecosystem by transferring stool from a screened healthy donor to the recipient's GI tract [82]. Although first validated for recurrent *Clostridioides difficile* infection, FMT has been explored for a range of disorders, including IBS, metabolic syndrome, and functional constipation [82].

Several clinical trials have reported encouraging results of FMT in STC. Xie et al. observed symptomatic improvement in 63% of patients and clinical remission in 75% after 3 treatment cycles, accompanied by shifts in the intestinal microbiota such as decreased Bacteroidetes and Firmicute but increased Actinobacteria and Proteobacteria [83]. Similarly, Zhang et al. demonstrated that co‐administration of soluble dietary fiber (pectin) with FMT augmented both short‐ and long‐term outcomes, achieving a clinical remission rate of 69.0% at week 4 and maintaining a success rate of 48.3% at the 1‐year follow‐up [84]. In addition, the delivery route of FMT may also influence its efficacy. In a previous study, the clinical improvement rate at 1 month after FMT was 74.2% for nasojejunal tube administration, 60.0% for oral capsule route, and 53.3% for colonoscopic infusion, respectively. The nasojejunal tube route achieved the best clinical efficacy, whereas oral capsules were more convenient, with less cost and higher patient acceptance [85]. A systematic review and meta‐analysis involving 245 adults—predominantly those with STC—reported that FMT achieved a pooled clinical remission rate (≥ 3 complete spontaneous bowel movements [CSBM] per week) of 50.7% (95% confidence interval [CI] 38.7%–62.7%) and an overall clinical improvement rate of 64.8% (95% CI 51.4%–76.3%), accompanied by significant improvements in Bristol Stool Form Scale score (mean difference [MD] 1.32), Wexner constipation score (MD −4.83), colonic transit time (MD −20.3 h), and GI quality‐of‐life index (GIQLI; MD 32.19) [86]. Moreover, post‐FMT microbial profiling in a subset of studies revealed increased α‐diversity and consistent enrichment of beneficial genera such as *Bifidobacterium*, *Prevotella*, *Fusicatenibacter*, and *Megamonas*, with concomitant suppression of proinflammatory *Enterobacteriaceae*. Adverse events were mild and transient, including abdominal bloating (in 17% of the cases), abdominal discomfort, and self‐limited diarrhea, and no serious complications occurred [86]. Despite these encouraging findings and a favorable short‐term safety profile, substantial heterogeneity (*I* ^2^ > 70%) and the predominance of single‐arm studies underscore the need for large, sham‐controlled randomized trials to confirm its long‐term efficacy and optimize protocols.

### 5.2 Acupuncture

Acupuncture, a component of traditional Chinese medicine, has been applied as an adjunctive treatment for STC aiming to enhance GI motility through modulation of the activities of sympathetic and parasympathetic nervous systems, neurotransmitter release, and smooth muscle contractility [87].

A multicenter RCT conducted by Liu et al. investigated the effects of electroacupuncture (EA) in 1075 patients with chronic severe functional constipation, a population in whom STC represents a physiological subtype, who were randomized to receive either 28 EA sessions at traditional acupoints or sham EA at non‐acupoints for over 8 weeks. The results showed that EA led to a significantly greater increase in weekly complete spontaneous bowel movements (CSBMs) than sham treatment, with improvements persisting during a 12‐week follow‐up [88]. Notably, over one‐third of EA‐treated patients achieved at least three weekly CSBMs during the follow‐up period, compared with only 14% of the control group. Adverse events related to acupuncture were infrequent, mild, and transient. Although this trial did not exclusively recruit patients with manometrically or scintigraphically confirmed STC, the magnitude and durability of motility improvement strongly suggest that EA may benefit individuals with reduced colonic transit [88]. Therefore, EA may act through modulation of autonomic pathways that enhance parasympathetic and attenuate sympathetic outflow, thereby improving distal colonic motility.

### 5.3 Sacral Nerve Stimulation (SNS)

SNS is a minimally invasive and reversible modality that is currently recognized as effective for treating patients with urinary or fecal incontinence stemming from various causes, which has yielded positive long‐term results. It has also shown promising effects on the amelioration of other pelvic floor dysfunctions such as constipation [89]. A systematic review including 13 studies on SNS for chronic constipation has shown that test stimulation succeeded in 42% to 100% of the patients. In addition, up to 87% of the cases receiving permanent SNS achieved sustained symptom improvement during a median follow‐up of 28 months, with corresponding gains in QoL and patient satisfaction [90].

Studies have suggested that symptom improvement can be observed following SNS in patients with STC and obstructed defecation, with a success rate ranging from 22% to 73% depending on the length of follow‐up duration, regardless of the type of constipation [89]. However, study results regarding its effectiveness remain contradictory, largely due to the variations in study designs and population characteristics. SNS has been implicated in inducing colonic propagating contractions. Initial studies hinted at its potential benefit for patients with STC. However, a subsequent double‐blind, randomized, sham‐controlled trial, which represents a higher level of clinical evidence by controlling for placebo effects, has indicated that SNS is not correlated with improved constipation symptoms or an acceleration in colonic transit time [91]. Schiano di Visconte et al. enrolled 25 STC patients who underwent SNS test stimulation, showing a promising short‐term outcome after 6 months; however, there was a declining trend beyond this timeframe, raising questions about its long‐term efficiency [92]. In an RCT conducted by Dinning et al., 59 patients with refractory STC were randomized to receive either suprasensory/subsensory SNS or sham stimulations. The authors found that SNS did not enhance the frequency of complete bowel movements during the 3‐week active period [93]. In another RCT performed in the Netherlands, 67 patients with refractory idiopathic STC were treated either with SNS or pharmacological interventions. After 6‐month treatment, 54% of patients managed with SNS were successfully treated, compared to only 4% of those managed with pharmacological interventions, with those treated with SNS reporting lower severity of constipation and fatigue scores, along with an improved QoL. Notably, only eight serious adverse events were reported, with six occurring in the SNS group [94].

A recent systematic review on the effectiveness, safety, and cost‐effectiveness of SNS for idiopathic STC has revealed divergent findings regarding the efficacy of sacral neuromodulation (SNM). The infection rates ranged from 0% to 22%, while reoperation rates varied between 0% and 29%. Furthermore, whether SNM is cost‐effective or not compared to personalized conservative treatment after 6‐month treatment remains inconclusive [95]. In general, the efficacy of transcutaneous electrical stimulation for treating STC is uncertain, though it could offer benefits for certain medically refractory cases and is considered safe with no significant adverse events. Analogously, a meta‐analysis of exclusively RCTs encompassing 187 patients with predominantly STC went a step further by demonstrating no statistically or clinically meaningful superiority of SNM over sham stimulation or conservative therapy regarding constipation relief (odds ratio [OR] 1.92, 95% CI 0.68–5.42), Wexner score reduction, bowel movement frequency, or QoL [96]. Sensitivity analyses confirmed that any apparent benefit vanished when SNM was compared strictly against sham stimulation, showing that SNM offers no tangible improvement beyond control treatment and to question its routine application outside highly selected cases [96].

Transabdominal electrical stimulation has been demonstrated to increase colonic propagating pressure waves, albeit its investigation has been limited to pediatric STC cases [97]. Furthermore, colonic pacing utilizing intramuscular electrode placement, as an experimental approach for treating STC, has shown some promise in preclinical studies [98]. Further studies are warranted before its application can be endorsed.

In summary, current evidence indicates that whether SNS is superior to sham stimulation or conventional pharmacological interventions in treating STC remains controversial. Therefore, it should not be routinely recommended and may be considered only in highly selected refractory STC patients.

### 5.4 Transanal Irrigation (TAI)

TAI involves the controlled instillation of lukewarm water into the rectum and distal colon via a catheter or cone device to stimulate evacuation of feces [99]. It can be performed at home and is used primarily in neurogenic bowel dysfunction but has also been trialed in chronic constipation including STC. TAI protocols typically involve instilling 500–1000 mL of water, which is recommended to begin at a daily frequency, and reduce to alternate days after 10 to 14 days, with adjustment of the frequency according to patient tolerance and stool consistency [99]. TAI promotes reflex colonic contractions and facilitates complete rectal emptying. Potential advantages of the procedure include noninvasiveness, avoidance of systemic medication‐associated side effects, and compatibility with home‐based regimens.

However, adherence to TAI in STC remains low [99]. A previous study has revealed that only 40% of patients continued TAI beyond 12 months, with discontinuation most commonly due to perceived lack of benefit, procedural inconvenience, and discomfort [100]. Minor adverse events such as transient abdominal cramps, leakage, or catheter‐related irritation are relatively common, while serious complications (e.g., bowel perforation) are rare, usually in the context of inappropriate technique or preexisting pathology.

In summary, TAI may be used as a bridging therapy for patients who have failed oral and behavioral management but are not ready for or unsuitable for invasive surgical modalities. Optimal outcomes require patient training, individualized scheduling, and regular follow‐up to reinforce adherence. Its role in STC remains adjunctive rather than definitive, pending further controlled trials assessing long‐term efficacy and QoL impact. Non‐pharmacological therapies for STC increasingly reflect a shift toward mechanism‐based and phenotype‐driven management. Robust RCTs are needed to clarify which patient subgroups derive sustained benefit from these targeted approaches.

## 6 Endoscopic or Surgical Interventions for Refractory STC

### 6.1 Antegrade Continence Enema (ACE)—The Malone Procedure

In refractory STC cases, endoscopic or surgical interventions may be considered. Among them, ACE may be a preferred option to ambulatory colectomy [11]. Before the establishment of cecostomy or appendicostomy, which can be achieved via percutaneous endoscopic cecostomy or appendiceal and ileal conduit, antegrade colonic enema can be used to facilitate colonic emptying [11]. Selection between these two modalities depends on both the characteristics of the patient and the surgeon's preference, though appendiceal conduit is typically favored in pediatric cases. Compared to colectomy, they are less invasive, especially endoscopic cecostomy, which can be performed under local anesthesia and conscious sedation. They have been found to improve constipation symptoms in most patients [6]. Various irrigation solutions, such as tap water, saline, PEG, glycerin, and mineral oil, are utilized for ACE [11]. King et al. examined 56 pediatric STC cases treated with appendicostomy and revealed an 81% success rate with ACE. Among the 42 children presenting with abdominal pain, 37 experienced decreased severity and frequency of abdominal pain, with minimal long‐term complications such as granulation tissue, stomal infection, and washout leakage, and high satisfaction reported by families [101]. Another study investigated the long‐term outcomes of the ACE procedure for constipation in adults, showing that satisfactory ACE function was achieved in 47% of patients with STC over a median follow‐up of 36 months. A significant proportion (88%) of patients required at least one additional procedure on the primary conduit, with conduit reversal being necessary for 59% of them. Among the various conduit types, ileal conduit was associated with fewer minor revision procedures for stenosis when compared to appendix conduit. Notably, only three patients ultimately underwent colectomy [102]. In addition, Husťak et al. documented that laparoscopic‐assisted percutaneous endoscopic cecostomy (LAPEC) achieved a treatment success rate of 60% in adults with severe STC, and offered both endoscopic and laparoscopic visualization, thereby reducing risks by ensuring accurate and safe access to the cecum and minimizing potential complications [103].

Notably, studies on the efficacy of ACE in refractory adult STC cases remain limited. Patients who might otherwise require colectomy may benefit from this approach, and the creation of cecostomy/appendicostomy does not seem to preclude them from potential future surgeries.

### 6.2 Surgical Decision‐Making

Surgical intervention is rarely indicated for patients with constipation, as it should be considered only in those with refractory STC in whom secondary reversible causes have been ruled out and pharmacologic treatment options have been ineffective [49]. Although the criteria for patient selection are crucial, when appropriate, patient outcomes can be favorable with significantly improved symptoms. In addition, surgery might also be necessary for certain patients to address anatomical abnormalities like stenotic diverticulitis or outlet disorders that may induce impaired defecation in the absence of STC.

Surgical options for treating STC mainly include ileostomy, total colectomy with ileorectal anastomosis, subtotal colectomy with cecorectal anastomosis, and modified Duhamel procedure [49, 104, 105]. For patients at a high risk for surgery, ileostomy without colectomy may be considered. Segmental colectomy has also been used, though the functional results and postoperative QoL satisfaction remain debatable [106, 107].

For cases with combined STC and dyssynergic defecation and STC, treatment of dyssynergic defecation should be prioritized before considering definitive surgery. If pelvic floor dysfunction cannot be corrected, ileostomy is the only surgical intervention leading to symptom relief [108].

#### 6.2.1 Total, Subtotal or Segmental Colectomy and Anastomosis

Total or subtotal colectomy remains the most commonly adopted surgical intervention for patients with objectively documented, medically refractory, isolated STC after excluding coexisting dyssynergic defecation or pelvic floor dysfunction [109]. In selected patients it can be highly effective, whereas poor candidate selection is the main reason for unfavorable outcomes.

Tian et al. demonstrated that 93.1% of the patients who underwent subtotal or total colectomy reported beneficial outcomes at 2 years post‐operation, presenting as a significant increase in the number of bowel movements per week, accompanied by notably improved symptoms such as bloating and straining, and decreased use of laxatives and enemas throughout the follow‐up period [110]. In addition, hemicolectomy is suggested as an alternative to colectomy with ileorectal anastomosis with a good short‐term outcome but requires thorough investigation and preoperative planning to determine the affected part of the bowel that needs to be excised. Tsimogiannis et al. investigated the efficacy of hemicolectomy for treating refractory STC in 50 patients, and reported that after a 20‐year follow‐up, 13 patients experienced treatment failure necessitating either additional surgery or irrigation. The authors concluded that hemicolectomy significantly increased stool frequency and reduced the need for laxatives, with a long‐term success rate ranging from 34% to 70%, depending on the outcomes in nonresponders [107]. Zhong et al. investigated 49 STC cases who underwent either laparoscopic subtotal colectomy or laparoscopic selective colectomy, targeting the specific area identified by barium‐strip technique, showing no significant differences in their postoperative complications, spontaneous bowel movements, and the Wexner Constipation Scale between the two groups. However, the authors noted that those who underwent laparoscopic selective colectomy achieved a significantly better GIQLI [106].

**FIGURE 2:**
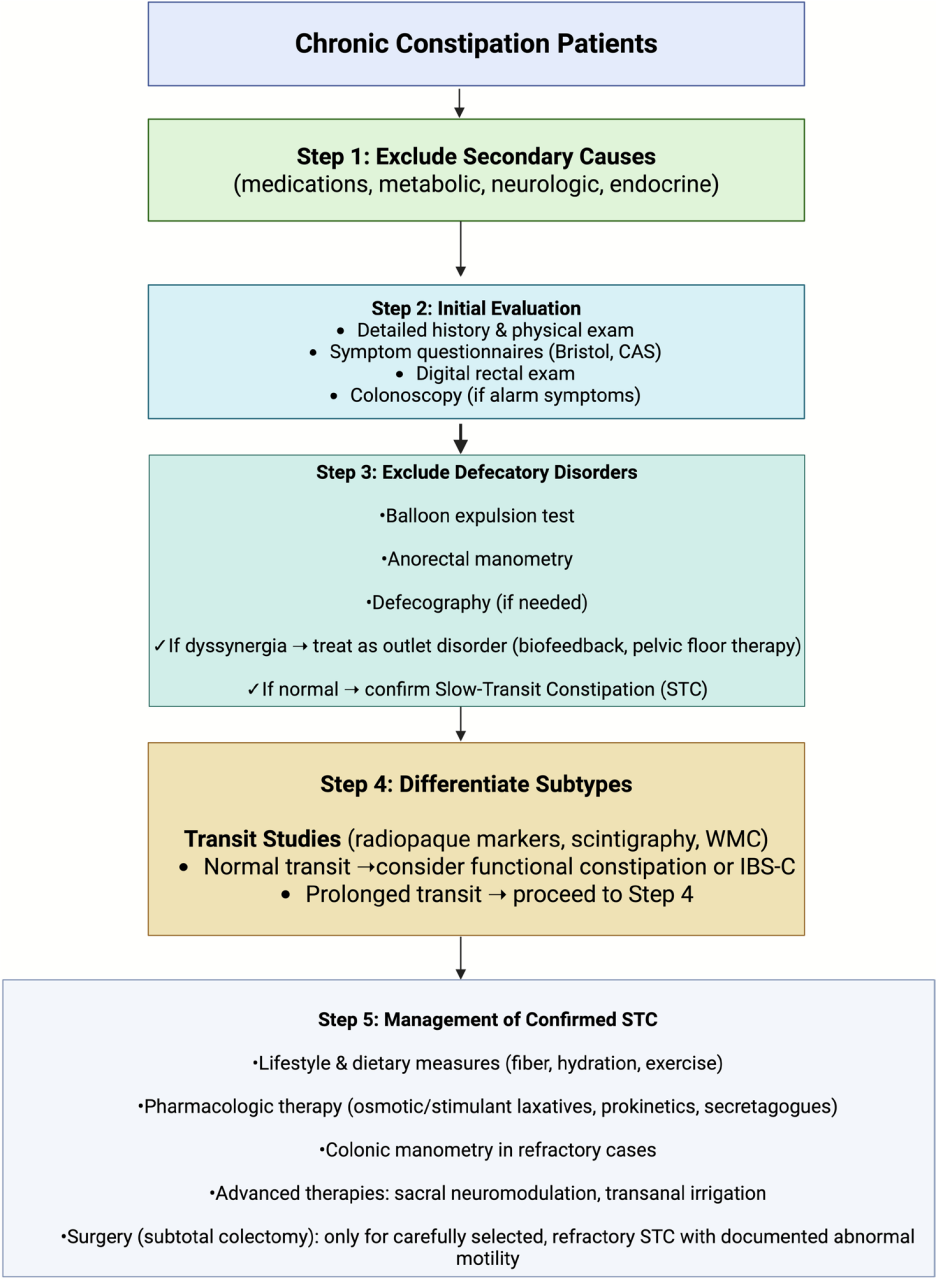
Recommended diagnostic and management algorithm for chronic constipation emphasizing the importance of excluding defecatory disorders (i.e., dyssynergic defecation) with anorectal physiology testing before performing colonic transit studies and diagnosing isolated slow‐transit constipation (STC). Escalating therapies specific to STC should only be considered in patients with objectively proven slow transit and normal pelvic floor function. CAS, constipation assessment scale; IBS‐C, irritable bowel syndrome with predominant constipation; WMC, wireless motility capsule. Created with BioRender.com.

A recent network meta‐analysis compared the effects of different colectomies and anastomoses on short‐term postoperative complications and QoL in patients with refractory STC [111], which revealed that subtotal colectomy with cecorectal anastomosis was highly effective in several key areas, including shorter length of hospitalization, reduced operative time, lower constipation indices, and improved QoL. In addition, regarding short‐term clinical outcomes, subtotal colectomy with ileosigmoidal anastomosis outperformed other modalities in reducing small bowel obstruction, alleviating abdominal pain and distension, and lowering incision infection rates. Moreover, subtotal colectomy with ileosigmoidal anastomosis ranked the highest for patient satisfaction. Based on these findings, cecorectal anastomosis is recommended for patients with refractory STC [111]. Deng et al. evaluated the clinical effectiveness of laparoscopic subtotal colectomy with antiperistaltic cecorectal anastomosis in patients with STC [112]. The authors demonstrated no significant differences in postoperative Wexner scale and GIQLI between the two groups. Though both anastomosis techniques were effective and safe for treating adult STC cases and significantly enhance patients' QoL, more patients reported satisfaction with defecation frequency following cecorectal anastomosis at 3 months post‐surgery. Moreover, despite cecorectal anastomosis showing better early outcomes in terms of defecation frequency, antidiarrheal usage, and overall satisfaction, the long‐term follow‐up results indicated similar outcomes for both procedures [112]. To reduce postoperative leakage rates following ileorectal anastomosis, Pu et al. sought to preserve the superior rectal artery in patients undergoing laparoscopically assisted subtotal colectomy with ileorectal anastomosis for STC and found that preserving the superior rectal artery was associated with improved postoperative bowel function recovery and lower rates of anastomotic leakage [113]. The key steps in the diagnostic and therapeutic approach to STC are summarized in Figure 2.

## 7 Conclusions and Future Perspectives

STC is a heterogeneous neuromuscular disorder rather than a single entity [108]. Across published cohorts, patients share a characteristic reduction in propulsive activity—particularly HAPCs—with variable contributions from impaired pacemaker networks (interstitial ICCs) [28], enteric neuropathy, autonomic imbalance [36], and modulators such as bile acids [52], methane, and the intestinal microbiota [49]. Because delayed colonic transit may also accompany defecatory disorders, diagnosis must be anchored in objective transit testing and a careful anorectal evaluation to exclude pelvic floor dysfunction before the label of “true” STC is applied [49].

Management of STC should remain stepwise and phenotype‐driven [5, 48]. Lifestyle measures (fiber—preferably soluble—and activity) and osmotic or stimulant laxatives are first‐line treatment options. While secretagogues and the selective 5‐HT_4_ receptor agonist offer clinically meaningful gains for many but not all patients [48]. When dyssynergia coexists, anorectal biofeedback is the preferred therapy and often changes the natural history [48]. Interventional options, including FMT, acupuncture, SNS, and TAI, are promising for selected refractory cases but remain variably supported and should be framed as adjunctive or investigational pending larger, well‐controlled trials [104]. For a minority with rigorous documentation of isolated colonic motor failure (ideally with manometry and/or histology), surgery can be effective [104]. Among operative strategies, subtotal colectomy with reconstruction tailored to patient factors remains the most established, while antegrade continence enemas provide a reversible bridge in carefully chosen cases [109].

Looking ahead, progress will depend on: (i) more precise phenotyping that integrates transit profiling, high‐resolution manometry, and biomarkers (microbiome, bile acids, methane); (ii) standardized, patient‐centered outcomes beyond stool frequency; and (iii) targeted therapeutics that move beyond global laxation to restore motility circuits—whether via enteric neurodegeneration, ICC support, bile acid manipulation, or microbiome‐directed approaches. Multidisciplinary care that couples gastroenterology, pelvic floor physiology, surgery, nutrition supplementation, and psychology remains essential to align treatment intensity with pathophysiology and patient goals. Future research should focus on standardized phenotyping and multicenter trials that integrate biomarkers (e.g., microbiome, bile acids, methane) with physiological testing. Well‐designed RCTs and prospective surgical registries are needed to refine patient selection and guide precision, mechanism‐based therapies for STC.

## Funding

No funding was received for this work.

## Conflicts of Interest

The authors declare no conflicts of interest.

## Data Availability

Data sharing not applicable to this article as no datasets were generated or analyzed during the current study.
